# The influence of Stigma, Disclosure, and Self-Esteem on Quality of Life in Individuals with Mental Illness

**DOI:** 10.1192/j.eurpsy.2025.1487

**Published:** 2025-08-26

**Authors:** Y.-J. Lien, Y.-H. Wang

**Affiliations:** 1 National Taiwan Normal University, Taipei, Taiwan, Province of China

## Abstract

**Introduction:**

Stigma not only influences the willingness to disclose mental health conditions and self-esteem but may also diminish the overall quality of life in individuals with mental illnesses. However, limited research has examined the potential mechanisms underlying this complex relationship.

**Objectives:**

This study aims to explore the mediating roles of disclosure and self-esteem in the association between mental illness stigma and quality of life.

**Methods:**

We utilized the meta-analytic structural equation modeling (MASEM) approach and conducted a comprehensive literature search across various electronic databases to identify relevant publications up to July 2023. MASEM was employed to derive bivariate correlation matrices for stigma, disclosure, self-esteem, and quality of life. Additionally, two simple mediation models and one serial mediation model were tested to examine the relationships between these variables.

**Results:**

The analysis included 181 articles reporting 195 independent samples (N = 33,162) and 278 effect sizes. The single mediator model indicated that self-esteem (β = −0.155, 95% CI [−0.276, −0.070], p < .001), rather than disclosure (β = −0.019, 95% CI [−0.094, 0.031], p > .05), served as a mediator. In the multiple mediator model, disclosure and self-esteem were found to have serial mediating roles between stigma and quality of life (β = −0.016, 95% CI [−0.0546, −0.0003], p < .05).

**Conclusions:**

This study makes a significant contribution to understanding how stigma attitudes impact the quality of life in individuals with mental health problems, providing a strong empirical foundation for the development of mental health interventions. Future research directions and practical implications are also explored.

**Disclosure of Interest:**

None Declared

